# Evaluating the effects of a multi-level intervention on young women’s contraceptive use: sexual and reproductive health results of a cluster-randomised factorial-design study in Tshwane, South Africa

**DOI:** 10.1080/26410397.2026.2696689

**Published:** 2026-07-01

**Authors:** Ilene S. Speizer, Courtney P. Bonner, Laura Nyblade, Jacqueline Ndirangu, Alexandra Minnis, Khatija Ahmed, Tracy L. Kline, Felicia A. Browne, Brittni Howard, Wendee M. Wechsberg

**Affiliations:** a Professor, Department of Maternal and Child Health, University of North Carolina at Chapel Hill, Chapel Hill, NC, USA; Faculty Fellow Carolina Population Center, University of North Carolina at Chapel Hill, Chapel Hill, NC, USA. *Correspondence*: ilene_speizer@unc.edu; b Senior Research Psychologist, RTI International, Durham, NC, USA; c Fellow and Senior Technical Advisor, RTI International, Durham, NC, USA; d Senior Public Health Analyst, RTI International, Durham, NC, USA; e Public Health Director, RTI International, Durham, NC, USA; f Investigator, Setshaba Research Centre, Tshwane City, South Africa; Researcher, Department of Paediatrics & Child Health, in the School of Clinical Medicine, University of the Witwatersrand, Johannesburg, South Africa; g Senior Manager Research Psychometrician, RTI International, Durham, NC, USA; h Senior Research Social Epidemiologist, RTI International, Durham, NC, USA; i Public Health Analyst III, RTI International, Durham, NC, USA; j Adjunct Professor, Department of Maternal and Child Health, University of North Carolina at Chapel Hill, Chapel Hill, NC, USA; Principal Investigator Emerita, RTI International, Durham, NC, USA

**Keywords:** adolescent girls and young women, contraceptive use, stigma and discrimination, intervention, evaluation, South Africa

## Abstract

In South Africa, adolescent girls and young women (AGYW) are engaged in early, condomless, and unprotected (from pregnancy) sex, which puts them at risk of unintended pregnancies and sexually transmitted infections. At the individual, interpersonal, and structural levels, AGYW experience barriers to their access to and use of contraception and condoms. This study employed a cluster-randomised factorial design to examine the impact of a multi-level intervention on contraceptive use behaviours among 802 women ages 16–24 in Tshwane, South Africa. The two intervention components were the Young Women’s Health CoOp (YWHC), an intervention to increase participants’ knowledge and skills, and a stigma and discrimination (S&D) reduction training at the facility level. Study facilities were randomised into the four study arms: YWHC only; S&D only; both YWHC and S&D; and control (standard of care). The study demonstrated that AGYW in the YWHC arm (RRR: 2.45; 95% CI: 1.05–5.70, *p* = 0.038) and those in the YWHC and S&D arm (RRR: 3.65; 95% CI: 1.56–8.50, *p* = 0.003) were more likely to have started a contraceptive method than to remain a non-user compared to those in the control arm over the 9-month follow-up period. However, those in the S&D training-only arm had lower odds of adopting a method over the follow-up period (OR: 0.63; 95% CI: 0.41–0.97, *p* = 0.037). These results demonstrate the importance of supporting AGYW with tailored messaging in a safe environment as part of sexual and reproductive health services.

DOI: 10.1080/26410397.2026.2696689

## Plain language summary

In South Africa, many teenage girls and young women start having sex at a young age, often without using condoms or birth control. This puts them at risk of unintended pregnancy and sexually transmitted infections. This study looked at young women ages 16–24 in Tshwane, South Africa, and tested a programme to help them use birth control and HIV prevention more often. The programme had two parts: the Young Women’s Health CoOp (YWHC), which worked directly with young women to teach and support them, and training for clinic staff to reduce stigma and discrimination and improve how they treat young women. The study included four groups: YWHC only, staff training only, both YWHC and staff training, and a control group that received usual care. The results showed that young women who took part in the YWHC programme, and those who received both parts, were more likely to start using birth control and to keep using it regularly over the 9-month follow-up period. In contrast, young women in the group with only staff training were less likely to start or use birth control during this time. These findings show that it is important to provide programmes that directly support young women in a safe and respectful environment and address their needs for sexual health, pregnancy prevention, and HIV prevention.

## Résumé

En Afrique du Sud, les adolescentes et les jeunes femmes ont des relations sexuelles précoces, sans préservatif ni protection contre les grossesses, ce qui les expose à des grossesses non désirées et à des infections sexuellement transmissibles. Aux niveaux individuel, interpersonnel et structurel, elles rencontrent des difficultés pour obtenir et utiliser des méthodes de contraception et des préservatifs. Cette étude a utilisé une conception factorielle aléatoire par grappes pour examiner l’impact d’une intervention à plusieurs niveaux sur les comportements d’utilisation de la contraception chez 802 femmes âgées de 16 à 24 ans à Tshwane, en Afrique du Sud. L’intervention comportait deux volets : la Young Women’s Health CoOp (YWHC), une initiative visant renforcer les connaissances et les compétences des participantes, et une formation à la réduction de la stigmatisation et de la discrimination (S&D) dispensée dans les centres de santé. Les établissements participant à l’étude ont été répartis de manière aléatoire en quatre groupes : YWHC seulement ; S&D uniquement ; YWHC et S&D ; et groupe témoin (norme de soins). L’étude a démontré que les adolescentes et les jeunes femmes du groupe YWHC (RRR : 2.45 ; IC 95% :1.05–5.70, *p* = 0.038) et celles du groupe YWHC et S&D (RRR :3.65 ; IC : 95% : 1.56–8.50, *p* = 0.003) étaient plus susceptibles d’avoir commencé à utiliser une méthode contraceptive que de rester non-utilisatrices, comparativement au groupe témoin, au cours de la période de suivi de neuf mois. Néanmoins, les participantes du groupe S&D uniquement avaient moins de probabilités d’adopter une méthode contraceptive au cours de cette période de suivi (RC :0.63 ; IC 95% : 0.41–0.97, *p* = 0.037). Ces résultats montrent combien il est important d’aider les adolescentes et les jeunes femmes avec des messages adaptés, dans un environnement sûr, au sein de services de santé sexuelle et reproductive.

## Resumen

En Sudáfrica, las adolescentes y mujeres jóvenes (AMJ) participan en actos sexuales sin condón y sin protección (del embarazo), lo que las pone en riesgo de tener embarazos no intencionales e infecciones de transmisión sexual. A nivel individual, interpersonal y estructural, las AMJ enfrentan barreras de accesibilidad y uso de anticonceptivos y condones. Este estudio empleó un diseño factorial aleatorizado por conglomerados para examinar el impacto de una intervención multinivel en el uso de anticonceptivos y en los comportamientos relacionados con la anticoncepción entre 802 mujeres de 16 a 24 años, en Tshwane, Sudáfrica. Los dos componentes de la intervención eran la Cooperativa de Salud de Mujeres Jóvenes, intervención para aumentar los conocimientos y las habilidades de las participantes, y una capacitación para la reducción de estigma y discriminación (EyD) en centros de salud. Los centros del estudio fueron asignados al azar a cuatro grupos del estudio: AMJ solamente; EyD solamente; AMJ y EyD; y control (nivel de atención). El estudio demostró que las AMJ en el grupo de AMJ (RRR: 2.45; IC de 95%: 1.05–5.70, *p* = 0.038) y aquéllas en el grupo de AMJ y EyD (RRR: 3.65; IC de 95%: 1.56–8.50, *p* = 0.003) eran más propensas a iniciar un método anticonceptivo que a permanecer como no usuarias, comparadas con aquéllas en el grupo de control durante el período de seguimiento de 9 meses. Sin embargo, aquéllas en el grupo de capacitación en EyD solamente tenían menos probabilidades de adoptar un método durante el período de seguimiento (RM: 0.63; IC de 95%: 0.41–0.97, *p* = 0.037). Estos resultados demuestran la importancia de apoyar a las AMJ con mensajes dirigidos a ellas, en un entorno seguro, como parte de los servicios de salud sexual y reproductiva.

## Introduction

In South Africa, many adolescent girls and young women (AGYW) are having early condomless and unprotected (from pregnancy) sex, which puts them at risk of unintended pregnancies and sexually transmitted infections. Results of the 2016 South Africa Demographic and Health Survey (DHS) showed that 3.1% of adolescents (ages 15–19) and 19.2% of young women (ages 20–24) were married or in union. Furthermore, 43% and 92% of women aged 15–19 and 20–24, respectively, had had sex. Among these sexually active young women aged 15–24, only about 60% reported using a modern method of contraception such as condoms, injectables, and implants; 38% reported engaging in condomless sex with a non-marital partner at last sex.^1^ In a more recent survey from six districts of South Africa involving nearly 4400 AGYW, the authors found that 69% had ever had penetrative sex with a median age of first sex of 20.^2^ In this study that focused on first sex experiences, the authors found that about 62% used a condom at first sex, and only about 23% used a modern contraceptive method other than condoms.^2^ The authors show that those who had an earlier sexual debut were more likely to have been coerced than those who had a later sexual debut.^2^ At the time of the 2016 DHS survey, nearly 16% of adolescent girls (aged 15–19) had begun childbearing (i.e. were already mothers or were currently pregnant), and only 21% of these girls reported that the pregnancies were desired at the time of conception.^1^ Further, a recent estimate of pregnancies in the public sector in South Africa demonstrates that the adolescent pregnancy rate (i.e. number of pregnancies to 10–19-year-olds per 1000) has been on the rise from 2017/18, when it was 26.8 per 1000, to 2021/22, when it was 30.5 per 1000.^3^

AGYW constitute a priority population for HIV prevention, accounting for 25% of new HIV infections; this incidence is three times higher than that among their male counterparts.^4^ The risk of HIV acquisition increases significantly in late adolescence and early adulthood; estimates from some parts of South Africa indicate that the prevalence goes from 4% among 15- to 19-year-olds to 24% among their 20- to 24-year-old counterparts.^4^

Collectively, these data indicate the need to ensure that AGYW have the autonomy and self-determination to access sexual and reproductive health information and services that support their sexual and reproductive health (SRH) and well-being. Importantly, access to and use of SRH services stands to empower AGYW to continue their schooling and advance their well-being through avoiding adverse health and social consequences, including unintended pregnancies, maternal and infant death, unsafe abortion, rapid repeat pregnancies, and sexually transmitted infections, including HIV.^5^

While it is imperative for AGYW to have the ability to use effective contraception, studies from South Africa demonstrate multi-level barriers that affect their right to available, accessible, acceptable, and high quality SRH services. These barriers exist at the individual, interpersonal and structural levels. At the individual level, AGYW’s lack of information on contraception side effects, HIV testing experience, perceptions of risk of HIV or a pregnancy, and peer influences affect their autonomy and ability to access or use condoms or contraception.^6–10^ At the interpersonal level, some AGYW face opposition to birth control or condom use from male partners or other family members.^9–11^ At the structural level, inconsistent policies in South Africa around age of consent and access to SRH services result in inconsistent access to contraception for young people, depending on the interpretation and comfort of providers and facility managers.^12^ An additional key barrier to South African young people’s use of SRH services, including HIV prevention, is stigma and discrimination (S&D) that they fear or experience from service providers.^10,13–15^

Many interventions and evaluations of SRH programmes for AGYW in South Africa focus on young people’s risk for HIV (i.e. delaying sexual debut and condom use)^16^ and more recently include promotion and adoption of pre-exposure prophylaxis (PrEP) for HIV prevention.^17,18^ AGYW, who are at increased risk of HIV compared to their male counterparts, are also vulnerable to factors that contribute to unintended pregnancies.

While there are not many evaluation studies of interventions to address SRH needs for adolescents in South Africa, besides HIV prevention, a recent scoping review of programmes that led to increases in contraceptive use from sub-Saharan Africa demonstrated some strategies that were found to be more effective.^19^ The authors demonstrate that community-based programmes, mHealth, sexual and reproductive health education, counselling, community health worker engagement, and youth-friendly health service interventions were associated with increases in contraceptive knowledge and use across diverse populations and settings. The one programme from the scoping review from South Africa was a mass media project that examined the impact of MTV Shuga on various HIV outcomes as well as uptake of contraception.^20^ The authors demonstrate that self-reported exposure to the MTV Shuga programme was associated with contraceptive uptake and consistent condom use.^20^ While the findings were positive, overall exposure to the mass media programme was low and the authors propose the need for multi-component programmes that also address other barriers to contraceptive use and HIV prevention strategies, including addressing provider stigma.^20^

Interventions are needed to address factors that may increase risk, as well as factors that may be protective against HIV and unintended pregnancy among AGYW. Young people often lack the agency and autonomy needed to meet their sexual and reproductive health needs, increasing their risk of unintended pregnancy or HIV^21^; programmes that address AGYW’s ability to make and act on sexual and reproductive health decisions can begin to support their human right to attain their personal goals and bodily autonomy.^22^ This study examined the impact of a multi-level intervention on contraceptive use and HIV-prevention behaviours among AGYW. The two main components of the intervention were (a) a group-level intervention that offered individuals information, skills building for sexual protection, and referral for provision of PrEP and SRH services, and (b) stigma and discrimination (S&D) reduction training at the facility level to address provider bias that is commonly experienced by young women seeking SRH services.^23^ This study examines the effect of each of these intervention components separately and jointly to understand the impact of each component and the multi-level intervention on contraceptive adoption and use among AGYW.

## Methods

### Intervention arms

This study examines the impact of two types of interventions separately and jointly on AGYW’s adoption and use of contraception, with the goal of determining whether intervening at multiple levels leads to better outcomes than intervening at the individual or health facility levels separately. The first component of the intervention was an evidence-based brief intervention, the Young Women’s Health CoOp (YWHC), that has previously been undertaken in Cape Town and found to have positive influences on the reduction of impaired sex (i.e. sex under the influence) and condomless sex.^24^ The YWHC focuses on addressing AGYW’s individual- and interpersonal-level factors that have been shown to affect adolescent sexual and reproductive health behaviours and outcomes.^6–11^ The YWHC is a two-session, four-module programme that engages young women through group discussions led by project field staff and undertaken under a tent at study clinics or in a private room at the project office. The first workshop, broken into two modules, focuses on becoming adults, sex and expectations, female reproductive bodies, STIs and HIV, and ways to reduce risk, learning about condoms and negotiating protective sex, PrEP and birth control, with each participant developing their own personal action plan. The second workshop, broken into two modules, addresses inequality and power, abuse and violence, safety for going out, alcohol and drug use, parenting, the importance of education, social support and PrEP use while working on more goals for their individual action plan.^23^ For this project, the YWHC was adapted to the study context in collaboration with the project Community Collaborative Board (CCB) and the project Youth Advisory Board (YAB); adaptations included adding information, identifying appropriate local language and developing referral plans for PrEP and contraceptive use and other SRH needs.^23^ This type of tailored group-based intervention addresses young people’s agency and autonomy to seek health care services to meet their sexual and reproductive health needs. The educational aspects focus on reproductive body parts and types of birth control and address the risks and benefits of each method. In addition, the content addresses what alcohol and drugs do to people’s bodies and how to learn communication skills and prevent gender-based violence. The participants learn skills for greater sexual protection and what might be a healthier lifestyle by developing goals towards their future and by considering steps to achieve their goals.

The second component of the intervention addressed facility-level barriers that reduce AGYW's access to and use of SRH services, thereby addressing the right to universal healthcare for all. The focus of the intervention was on health worker stigma and discrimination towards AGYW seeking SRH services. For this component, the project adapted and implemented the Health Policy Project Health Facility HIV Stigma and Discrimination reduction training curriculum that has been widely implemented as part of the full Total Facility Approach to HIV stigma reduction,^25^ including in Ghana and Tanzania.^26–29^ For this study, this curriculum was modified to address health workers’ PrEP stigma and stigma towards AGYW seeking SRH services.^23^ It included three workshops with hands-on experiential exercises on topics such as naming stigma through pictures and experiencing stigma as the stigmatiser and stigmatised. The workshop addressed concerns about prescribing PrEP to AGYW and included a panel discussion with AGYW to understand their experiences with the goal of creating stigma-free services for youth. Health facility staff who were selected by their managers attended two 3-hour participatory training sessions that were delivered on consecutive days by master stigma reduction trainers from Zambia. For the first three S&D intervention clinics, the training was delivered in person, and health worker trainers received in-person coaching for the first two sessions they delivered. For the second set of intervention clinics, which took place after the COVID-19 pandemic, virtual training was conducted by the master stigma reduction trainers, and the training manual was revised to include social distancing as part of the training of the facility health care workers.

The Youth Advisory Board and Community Advisory Board were critical to the refinement of the study interventions (S&D and YWHC) and study questionnaires. We held meetings with these boards twice a year to inform them of the study progress and receive their input on study challenges. The Youth Advisory Board members also took part in the S&D training of trainers and in the S&D facility training activities.

### Study context and design

This study took place in the Tshwane metropolitan municipal area, which includes the major administrative city of Pretoria, South Africa. Gauteng Province,^1^ where Tshwane is located, had an HIV prevalence of 11.9% among people ages 25–49 years with prevalence more than ten points higher among females (24.9%) compared to males (14.1%).^30^ The City of Tshwane Health Department has called for heightened attention to the prevention of teenage pregnancy and the factors that are associated with the risk of adolescent pregnancy, including unsafe sexual practices, peer pressure, gender-based violence, drug and substance abuse, poverty and lack of knowledge about fertility, among others.^31^ Thus, this type of multi-component intervention is appropriate for reaching AGYW and meeting their varied sexual and reproductive health needs.

In Tshwane, the study team identified 15 provincial and city clinics to serve as the study clusters. Each clinic was visited to determine its eligibility. Clinic eligibility included (a) having administrative staff willing to participate; (b) having an outreach programme; (c) offering comprehensive SRH services, defined in the National Sexual and Reproductive Health and Rights Policy (NSRHRP) as having preventive, curative, and rehabilitative SRH services; and (d) providing youth-friendly services, having a youth-friendly staff person at the facility, or both. As defined in the NSRHRP, youth-friendly clinics or staff offer services that are both responsive and acceptable to adolescents and youth and are non-judgmental, confidential, offered in a private environment and at times convenient for young people.^2^ These minimum criteria were essential for implementing the two types of intervention components at the individual and facility levels and ensured minimum criteria for all facilities, including the control sites.^23^ While all 15 clinics met the eligibility criteria, an initial 12 clinics were selected for the study; the remaining three clinics served as back-ups should one or more of the recruited facilities decide not to participate. Twelve clinics were grouped into three strata defined by geographic location type (peri-urban vs. semi-rural) and clinic administrative level (city vs. provincial), which corresponded with clinic resources and patient volume. Within each stratum, clinics were randomly assigned to the four study arms based on a 1:1:1:1 allocation.

We estimated that the 12 clinics with 75 AGYW participants recruited per clinic (or cluster) provided the needed power to identify meaningful differences in the primary PrEP and SRH outcomes.^23^ Note that because of challenges and delays in implementation introduced during the COVID-19 pandemic, our final sample size in four of the 12 study clinics (one per arm) was reduced to 50 AGYW participants.^32^ Sample size estimates for tests of two proportions in a cluster-randomised design were conducted for a 2-sided test with significance level of 0.05, power of 0.80, and intra-cluster correlation of 0.01, assuming 10% attrition over the 9-month follow-up period. We were powered to detect a difference between 9 and 13% in primary PrEP and SRH outcomes, which is a realistic difference based on previous research.^33–36^

This study used a cluster-randomised, two-by-two factorial design to determine the impact of the YWHC and the S&D reduction training. Note that PrEP and SRH clinic services and referrals (i.e. the standard of care) were accessible in all four study arms, including the control arm. The four arms of the study were (a) the control arm (standard of care); (b) the YWHC-only arm (individual/interpersonal intervention); (c) the S&D training of facility staff only arm (facility-level intervention); and (d) both the YWHC and the S&D training arm (individual/interpersonal/facility-level intervention).^23^ The study employed a phased rollout process rather than engaging all study clusters at once.

In the catchment area of each study facility, young women ages 16–24 were recruited. Community outreach workers assisted with recruitment. Participants were identified through street outreach at places where AGYW congregate, and using referrals from the clinic or other community health workers. Eligibility criteria were as follows: (a) had condomless sex in the past 3 months with a male partner; (b) were not currently pregnant and were not seeking a pregnancy in the next 12 months; (c) were willing to consider taking a daily HIV prevention pill; (d) had not been a part of the formative research for this project; (e) were not taking part in other studies related to HIV prevention; (f) were living in the target community; (g) were not currently taking other drugs such as tuberculosis treatment; and (h) intended to stay in the target area for the next 12 months.^23^ Recruitment started about a year before the COVID-19 pandemic (April 2019); although it slowed during the pandemic, we were able to continue some recruitment when lockdowns were relaxed (between June and December 2020) and then continued active recruitment after COVID-19 lockdowns ceased fully (January–July 2021). We consider these as three study periods: pre-COVID-19, during COVID-19, and post-COVID-19. Notably, we recruited across all arms before COVID and after COVID; however, during COVID, most recruitment continued in clusters that were engaged before COVID, and new clinics were brought on only in the two arms that did not include the S&D reduction training. The distribution of participating AGYW by intervention group and COVID classification can be seen in Tables 1 and 2.

**Table 1.** Descriptive characteristics of the baseline sample that was followed at 3, 6, and 9 months

	Full sample (*n* = 802) %	Missing (*n* = 35) %	Analysis sample (*n* = 767) %
**Intervention group**			
No intervention (control)	24.94	37.14	24.38
YWHC only	27.93	31.43	27.77
S&D only	21.82	20.00	21.90
YWHC and S&D	25.31	11.43	25.95
**Baseline age**			
16–17	17.45	17.21	22.86
18	12.97	5.71	13.30
19	14.09	8.57	14.34
20	13.72	22.86	13.30
21	13.84	17.14	13.69
22	11.35	14.29	11.21
23–24	16.58	8.57	16.95
**Baseline relationship**			
Single	10.85	2.86	11.21
Has partner	87.66	97.14	87.22
Separated/divorced	1.50	0	1.56
**Baseline in-school status**			
No	49.75	45.71	49.93
Yes	50.25	54.29	50.07
**Baseline ever-pregnant status**			
No	58.35	71.43	57.76
Yes	41.65	28.57	42.24
**Baseline number of partners**			
1	62.75	69.7	62.45
2	20.63	15.15	20.86
3	10.25	12.12	10.17
4+	6.38	3.03	6.52
**Enrolment/COVID timing**			
Pre-COVID enrolment	46.76	51.43	46.54
During COVID	12.47	2.86	12.91
Post-COVID	40.77	45.71	40.55

Note: 33 observations were missing follow-up information at all time periods; 2 observations were missing a baseline number of partners.

**Table 2.** Descriptive characteristics of analysis sample by arm

	Control (*n* = 187) %	YWHC only (*n* = 213) %	S&D only (*n* = 168) %	YWHC and S&D (*n* = 199) %
**Baseline age**				
16–17	17.11	17.84	14.29	19.10
18	15.51	8.92	13.10	16.08
19	11.76	19.72	13.69	11.56
20	15.51	14.08	11.31	12.06
21	11.76	15.02	16.07	12.06
22	9.09	13.62	11.90	10.05
23–24	19.25	10.80	19.64	19.10
**Baseline relationship**				
Single	8.02	11.74	10.12	14.57
Has partner	89.84	86.38	89.29	83.92
Separated/divorced	2.14	1.88	0.60	1.51
**Baseline in-school status**				
No	50.27	52.58	47.02	49.25
Yes	49.73	47.42	52.98	50.75
**Baseline ever pregnant status**				
No	54.01	64.32	58.33	53.77
Yes	45.99	35.68	41.67	46.23
**Baseline number of partners**				
1	64.71	56.81	70.24	59.80
2	21.93	22.07	18.45	20.60
3	8.56	12.21	5.95	13.07
4+	4.81	8.92	5.36	6.53
Enrolment/COVID timing***				
Pre-COVID enrolment	37.43	54.93	39.29	52.26
During COVID	8.02	15.49	5.36	21.11
Post-COVID	54.55	29.58	55.36	26.63

***p < 0.001 indicates a significant difference across groups.

### Data collection methods

AGYW who were willing to participate were screened for eligibility and, if eligible, were asked to come to the study clinic for consenting and baseline data collection. All participants reviewed the consent form with the study field staff and provided written consent or assent (minors ages 16–17) to participate. For study participants who were under age 18, written consent was required from a trusted adult who was at least 25 years old via in loco parentis; this approach was approved by the South African Medical Association Research Ethics Committee (SAMAREC #026102; 11 January 2019). After consent, participants completed a survey using a computer tablet with audio computer-assisted self-interview in either English or Setswana. Baseline study visits took on average 60–90 minutes, including biological testing for drugs, HIV, and pregnancy.

The survey covered topics related to background demographic information, PrEP knowledge and interest, access to health care and services, alcohol and drug use, depression and anxiety, sexual history, transactional sex, condom negotiations, power and empowerment, HIV testing and risk, pregnancy, and contraceptive use, among other topics. This analysis focused on the outcomes related to contraceptive adoption and use. At baseline, participants were tested for HIV and pregnancy. At 3-month, 6-month, and 9-month follow-ups, the AGYW were asked to return to the clinic and consented to a follow-up interview and clinical screening. The follow-up survey protocol included the same content areas as baseline as well as information about PrEP adoption and continuation; the clinical screening was the same as baseline (i.e. with HIV and pregnancy testing) with the addition of biomarkers for exposure to PrEP. If a young woman tested positive for a pregnancy at a follow-up visit, she remained eligible to stay in the study but was referred to a health care provider for counselling about PrEP. In total, 802 young women were recruited across the study arms.

The CONSORT diagram for the overall study can be found in the supplementary files and demonstrates high engagement in the two workshops across the study arms. In terms of engagement, in the YWHC-only arm, 79% of AGYW received the first workshop and 72% received the second workshop, while in the YWHC and S&D arm, 85% and 79% received the first and second workshops, respectively.

### Measures

**Figure 1.**
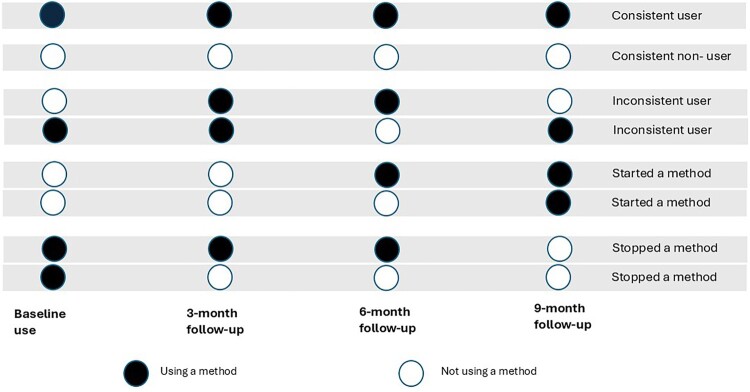
Classification of method use groups.

The project had two main outcomes: HIV prevention, including through PrEP adoption and continuation and condom use; and SRH outcomes, particularly related to contraceptive adoption and continuation. This paper focuses on the contraceptive use outcomes; another paper focuses on the PrEP outcomes.^32^ The main outcome variable of this analysis was contraceptive use. This was measured in two ways. First, based on all available information on modern contraceptive use^3^ reported at baseline and 3-, 6-, and 9-month follow-ups, we created an outcome that measured consistency of use. This outcome has five categories: (a) consistent non-user; (b) consistent user; (c) inconsistent user; (d) started a method (since baseline); and (e) stopped a method (since baseline). Figure 1 provides some of the different ways that a participant might be classified into these mutually exclusive groups. Note that consistent users may have changed the type of method used between baseline and a follow-up interview but were using a modern method at all study visits. The inconsistent users reported use at one or more time periods but were not consistent in their use behaviours. For example, inconsistent users may have been using at baseline and then reported periods of non-use and then reported use again at 9-month follow-up, or they may have started using at 3-month follow-up but not reported using at the other follow-up periods, among many other iterations. The group that “started a method” was not using at baseline but began using a method at any follow-up period and reported using a modern method at all subsequent follow-up periods. Note that some of the young women who “started a method” adopted only at 9-month follow-up. The “stopped” group was using a method at baseline and possibly at the 3- or 6-month follow-up but had stopped using at a follow-up visit and continued to not use at other follow-up visits; none of the “stopped” group was using a method at the 9-month follow-up visit. The distribution of the five consistency-of-use categories is shown in Table 3 by study arm. Given that an eligibility criterion was that the AGYW was not seeking a pregnancy in the next year, the intervention is meant to encourage AGYW to adopt or continue to use a method. Thus, programme “success” is observed if an AGYW is in the “consistent use” category or the “started a method” category. Notably, this definition of “success” is researcher-ascribed based on pregnancy desires and not based on an AGYW’s self-reported desire to use or not use a contraceptive method; we discuss the limitations of this approach in the discussion section of the paper.

**Table 3.** Contraceptive behaviours among AGYW at baseline and the pattern over the 9-month follow-up period by study arm

	Total (*n* = 767) %	Control (*n* = 187) %	YWHC only (*n* = 213) %	S&D only (*n* = 168) %	YWHC and S&D (*n* = 199) %
**Baseline method use**					
Non-user	49.93	48.66	48.83	57.14	46.23
Condom (male or female)	3.91	3.74	2.35	3.57	6.03
Oral contraceptive pill	8.34	10.70	7.51	8.93	6.53
Injectable	25.42	27.81	30.52	21.43	21.11
Implant/IUD	12.39	9.09	10.80	8.93	20.10
Contraceptive use pattern***					
Consistent non-user	16.82	19.25	11.74	29.76	9.05
Consistent user	26.73	25.67	30.99	16.67	31.66
Inconsistent user	20.08	18.72	19.72	19.64	21.61
Started a method	21.38	15.51	23.00	17.26	28.64
Stopped a method	14.99	20.86	14.55	16.67	9.05

***p < 0.001 indicates significant differences by study arm for contraceptive use patterns over the follow-up period.

The second contraception outcome created was whether the AGYW were using a modern method at each time period (yes or no). This outcome was used in the longitudinal analysis of contraceptive use over time and included all available information from the full sample, even for participants who did not contribute follow-up observations. Thus, information on this outcome comes from respondents who contributed anywhere from 1 to 4 observations, with an average of 3.6 responses per participant; baseline information from the small number of participants who did not continue in the study provides relevant information for grounding the arms.

The key independent variable for this analysis was the intervention arm. As mentioned above, the control group had no supplemental intervention beyond standard availability of PrEP and SRH services. The three intervention arms were created as categorical variables in the following groups: YWHC only, S&D reduction training only, and both YWHC and S&D training. These are compared to the reference group (the control arm).

### Covariates

A key determinant of contraceptive use is pregnancy experience. For the consistency-of-use analysis, we included a variable on whether the AGYW had ever been pregnant at baseline. In the longitudinal models, we included whether the AGYW tested positive for a pregnancy at the time of the follow-up data collection. Note that at each survey round, non-users were asked their reason for non-use of contraception. Fewer than 3% of respondents reported that they wanted to get pregnant as their reason for non-use; the main reason given was fear of side effects. Therefore, desire for a pregnancy was not included in study models.

Given that the intervention happened during COVID, it is important that we include variables on when the AGYW was recruited: before, during, or after COVID. For the longitudinal analysis, we also used the AGYW’s age at the time of interview round, her schooling status at the time of each interview, as well as her relationship status and the number of sexual partners in the previous 3 months at the time of each interview. The descriptive characteristics of the sample at baseline are shown in Tables 1 and 2.

### Analysis

Two types of analyses were performed for examining the impact of the interventions on AGYW contraceptive adoption and use. First, as mentioned above, we included a five-category outcome that assessed use patterns over the 9-month follow-up period. To evaluate the association between intervention arm and patterns of use, we used multinomial logistic regression and presented multiple comparisons. The main comparisons of interest were the first two shown in Table 4, which examined intervention effects on starting contraceptive method use (compared with those who remained consistent non-users) and on method discontinuation (compared with those who remained consistent users). The multinomial logistic regression analyses controlled for clustering at the clinic level and included key covariates and demographic factors described above. Results are presented as relative risk ratios.

**Table 4.** Multinomial logistic relative risk ratios (confidence intervals) of programme intervention arm on contraceptive behaviours over follow-up period, adjusting for clustering at the clinic level

	Started vs. Non-user	Stopped vs. Consistent	Inconsistent vs. Consistent	Started vs. Consistent	Non-user vs. Consistent
**Intervention group**					
No intervention	1.0	1.0	1.0	1.0	1.0
YWHC only	2.45* (1.05–5.70, *p* = 0.038)	0.60 (0.28–1.26, *p* = 0.179)	0.79 (0.26–2.35, *p* = 0.670)	1.10 (0.54–2.26, *p* = 0.794)	0.45^†^ (0.19–1.08, *p* = 0.074)
S&D only	0.73 (0.36–1.48, *p* = 0.380)	1.26 (0.78–2.02, *p* = 0.345)	1.64 (0.88–3.07, *p* = 0.120)	1.71 (0.76–3.87, *p* = 0.194)	2.36*** (1.62–3.43, *p* < 0.001)
Both	3.65** (1.56–8.50, *p* = 0.003)	0.37*** (0.23–0.60, *p* < 0.001)	0.91 (0.56–1.47, *p* = 0.689)	1.36 (0.71–2.58, *p* = 0.353)	0.37** (0.18–0.78, *p* = 0.009)
**Baseline pregnancy status**					
Never pregnant	1.0	1.0	1.0	1.0	1.0
Ever pregnant	2.12* (1.16–3.87, *p* = 0.014)	0.59* (0.37–0.94, *p* = 0.027)	0.45*** (0.29–0.70, *p* < 0.001)	0.60* (0.40–0.90, *p* = 0.013)	0.28*** (0.15–0.52, *p* < 0.001)
**COVID enrollment group**					
Pre-COVID	1.0	1.0	1.0	1.0	1.0
During COVID	2.13 (0.70–6.53, *p* = 0.184)	0.30* (0.11–0.81, *p* = 0.018)	0.66 (0.29–1.51, *p* = 0.329)	0.72 (0.44–1.17, *p* = 0.183)	0.34* (0.11–1.00, *p* = 0.050)
Post-COVID	0.80 (0.43–1.51, *p* = 0.493)	1.13 (0.71–1.80, *p* = 0.604)	0.95 (0.55–1.64, *p* = 0.859)	0.75 (0.46–1.22, *p* = 0.245)	0.93 (0.55–1.58, *p* = 0.791)

All models control for baseline age group, baseline relation, in school at baseline, and baseline number of partners. S&D is stigma and discrimination training.

n = 767; ^†^*p* ≤ 0.10; *p ≤ 0.05; **p ≤ 0.01; ***p ≤ 0.001. Model controls for clustering at the clinic level.

The longitudinal analysis, focusing on whether an AGYW was using at each time period (yes vs. no), used all available observations from the AGYW who participated in the study (n = 2,886, representing 802 women who contributed 1–4 observations each; the average was 3.6 observations). For this analysis, we used generalised estimating equations implemented in Stata statistical software with the *xtgee* command.^37^ We used the exchangeable correlation structure, a logit link, and binomial family, adjusting for clustering at the clinic level and including key covariates and demographic factors described above. Results are presented as odds ratios.

### Reflexivity statement

Our study, which engages AGYW from Tshwane neighbourhoods near provincial and city clinics, was developed with local partners in-country who have had a long-term partnership and collaboration with the lead research institution conducting women’s reproductive health and HIV research. In addition, a longstanding Community Advisory Board, as well as a Youth Advisory Board, guided the adaptation of the intervention and identified methods for recruitment. That said, the work is still shaped by the social position of the study team, which is organising advisory board meetings as well as leading the design of the study protocol. Our study team includes a diverse group of members, including one principal investigator who is a white woman from the United States (US) (WMW) who has worked in South Africa for two decades, and one who is a non-White South African based in Tshwane, South Africa (KA). The team also includes diverse co-investigators from Kenya (JN) and the US (ISS, CPB, LN, AM, TLK, FAB, BH), who have spent many years collaborating with colleagues on similar research and evaluation studies in sub-Saharan Africa, including South Africa. The research team brings perspectives from psychology, public health, and medicine and has worked across areas ranging from substance use, HIV prevention, sexual and reproductive health, gender, intimate partner violence, and maternal health. By identifying and training a team of local investigators and clinic providers to implement the intervention and to undertake the data collection, our full team represents the diversity of the population being served as part of the programme.

## Results

Table 1 presents the demographic characteristics of the full sample (n = 802), the sample missing any follow-up data (n = 33) or missing information on the number of partners (n = 2), and the final analysis sample for the consistency-of-use analyses (n = 767). About a quarter of the sample is in each of the intervention groups, as per the study design. As seen in Table 1, the analysis sample is spread across the age range 16–24. The overwhelming majority of the participants had a partner at baseline; no differences were found between the full sample and the analysis sample. At baseline, about half of the sample was currently in school. Two-thirds of the sample reported only one sexual partner in the previous 3 months and about a fifth reported two partners; 6% reported four or more partners in the previous 3 months. As discussed earlier, nearly half of the sample was enrolled before COVID, and a small number were enrolled during COVID; about two-fifths were enrolled after COVID. Table 2 presents the same demographic characteristics of the analysis sample by study arm. The number of observations by study arm is similar, with 187 in the control arm, 213 in the YWHC only arm, 168 in the S&D only arm, and 199 in the combined YWHC and S&D arm. The groups were generally comparable across the study arms, indicating that the randomisation across groups was largely successful. The one distinction found by study arm was that a greater percentage of the sample was from the post-COVID period in the S&D training arm and in the control arm; this reflects delays in training providers in some facilities during COVID.

Table 3 presents the baseline contraceptive use by study arm as well as the contraceptive use experiences over the follow-up period by arm. Nearly half of women in each study arm at baseline were non-users of a modern method of contraception. Among users, the main method used across all groups was injectable, followed by other long-acting methods such as implants and IUDs. In the examination of the five contraceptive use categories over the follow-up period, the largest group of women (more than a quarter) were consistent contraceptive users; that is, they were using a modern method at baseline and reported use at every follow-up visit. About a fifth of women started a modern method after baseline; another fifth were inconsistent users who reported multiple changes of stopping or starting over the study visits. Finally, about 17% of the young women in the study were consistent non-users over the course of the study, and another 15% were using at baseline but stopped by a follow-up visit.

Table 3 also demonstrates differences in the contraceptive use categories by study arm. More than 30% of AGYW in the YWHC only arm (30.99%) and in the YWHC and S&D arm (31.66%) were in the consistent user category whereas this category was lower in the S&D-only (16.67%) and control (25.67%) arms. The two arms that included YWHC also had a higher percentage of AGYW who started a method over the follow-up at 23.0% in the YWHC only arm and 28.64% in the combined YWHC and S&D arm; only 17.26% and 15.51% of women in the S&D only and control arms, respectively, started a method.

Table 4 presents the multinomial logistic regression coefficients and standard errors for the analysis of intervention effects on contraceptive use patterns. The first two comparisons are the most interesting, as they represent changes in status that might be related to intervention exposure. Those in the YWHC-only arm had more than two times the odds (RRR: 2.45; 95%CI: 1.05–5.70, *p* = 0.038) of starting a method than of remaining consistent non-users compared to those in the control arm. Furthermore, those who were in the joint intervention arm (both the YWHC and S&D training) had more than three times the odds of starting a method after baseline (RRR: 3.65; 95%CI:1.56–8.50, *p* = 0.003) compared to their counterparts in the control group. No effect on starting a method versus staying a consistent non-user was found among AGYW in clinic clusters in the S&D training-only arm.

Regarding intervention effects on contraceptive continuation, the comparison between those who stopped using contraception and consistent users provided evidence that those who were in the joint YWHC and S&D training arm had lower odds of stopping contraception than those in the control arm (RRR: 0.37; 95%CI: 0.23–0.60, *p* < 0.001). An intervention effect on contraceptive continuation versus stopping was not found for either intervention component offered alone.

No differences between arms were found between those who were inconsistent users and consistent users, and those who started a method and those who were consistent users. Finally, comparing the consistent non-users (i.e. non-users at baseline and other follow-up periods) to the consistent users, we see that those in the YWHC-only arm (RRR: 0.45; 95%CI: 0.19–1.08, *p* = 0.074) and in the combined intervention arm (RRR: 0.37; 95%CI: 0.18–0.78, *p* = 0.009) had lower odds of being non-users (i.e. more likely to be consistent users) than those in the control arm. Furthermore, AGYW in the S&D-only arm (RRR: 2.36; 95%CI: 1.62–3.43, *p* < 0.001) had higher odds than those in the control arm of being non-users than consistent users.

Table 4 also provides some of the other important independent variables and their associations with user groups. Those who were ever pregnant at baseline were more likely to have started a method after baseline than to be consistent non-users, and they were more likely to be consistent users than to be in any of the other groups (inconsistent, started, consistent non-user).

Timing of enrolment relative to COVID-19 was found to matter for two of the comparisons shown in Table 4. Those who enrolled during the COVID-19 shutdowns were more likely than those who enrolled before COVID-19 to be consistent users than to have stopped or to be non-users.

Table 5 shows the longitudinal model examining contraceptive use or non-use over the four time periods, adjusting for changing relationship status, number of partners, school status, age, and pregnancy experience. Here, we find that those young women who were in the S&D reduction training arm only had lower odds of using a modern method of contraception (OR: 0.63; 95%CI: 0.41–0.97, *p* = 0.037) than those in the control arm. That said, while the odds ratios were greater than one, no differences were found between those who were in the YWHC-only arm or the joint YWHC and S&D reduction training arm and the control group.

**Table 5.** Longitudinal model odds ratios (confidence intervals) examining modern method use over time by intervention group, adjusting for clustering at the clinic level

	Odds Ratio (95% CI)
**Intervention groups**	
No intervention	1.0
YWHC only	1.33 (0.86–2.05, *p* = 0.206)
S&D only	0.63* (0.41–0.97, *p* = 0.037)
Both	1.32 (0.85–2.05, *p* = 0.210)
**Longitudinal pregnancy experience during follow-up**	
No pregnancy	1.0
Pregnancy during follow-up	0.23*** (0.13–0.40, *p* < 0.001)
**COVID enrolment group**	
Pre-COVID	1.0
During COVID	1.44** (1.11–1.88, *p* = 0.007)
Post-COVID	1.04 (0.78–1.40, *p* = 0.783)

Model performed using xtgee in Stata statistical software with exchangeable correlation structure. S&D is stigma and discrimination training.

All models control for age, number of partners, in-school status, and relationship status at each of the time periods.

n = 767; †*p* ≤ 0.10; *p ≤ 0.05; **p ≤ 0.01; ***p ≤ 0.001. Model controls for clustering at the clinic level.

n = 2,886 (using data from all 802 women who have 1–4 observations, average is 3.6).

*p ≤ 0.05; **p ≤ 0.01; ***p ≤ 0.001.

Not surprisingly, young women who became pregnant over the follow-up period had lower odds of using any modern contraception method at each follow-up interval (OR: 0.23; 95%CI: 0.13–0.40, *p* < 0.001). Those who enrolled during the COVID-19 shutdowns were more likely to be using contraception than those who enrolled before COVID (OR: 1.44; 95%CI: 1.11–1.88, *p* = 0.007); no differences were found between those who enrolled before and after COVID.

## Discussion

This study examined the impact of an evidence-based, young woman-focused group-based intervention as well as an S&D reduction training at the facility level to address individual and facility-level barriers to young women’s adoption and use of contraception. The YWHC is an empowerment-based intervention that has previously been found to increase young South African women’s knowledge and skills to reduce substance use and increase self-efficacy for HIV preventive behaviours.^24^ This intervention was adapted in collaboration with a Community Collaborative Board and a Youth Advisory Board to include context-specific information as well as design local language and marketing approaches acceptable to AGYW. Our findings demonstrate that the YWHC arms of the intervention – that is, YWHC alone and in combination with the S&D reduction training – were associated with greater contraceptive use as measured by the pattern of contraceptive use outcomes, such that AGYW in the two intervention arms had higher odds of starting a method, continuing a method, and using a method consistently over the follow-up period. That said, the young women who were in the S&D reduction training arm only were not more likely to adopt, continue, or use contraception. In fact, in the longitudinal model, they were less likely than those in the control arm to be users over time.

The findings related to the S&D reduction training are counter to what was expected, given that the programme was meant to address a key barrier to young women’s SRH service use related to provider bias and mistreatment. Our findings of no effect of the S&D training on the key SRH outcomes may reflect that our implementation of the S&D training had a different scope and intensity than the original Health Policy Project’s (HPP) Total Facility Approach (TFA) intervention.^25^ Specifically, staff training was only one aspect of the more comprehensive original intervention, which in addition to other S&D reduction activities also had significant engagement of facility management and trained a larger proportion of facility staff than was possible in this study. In addition to being designed as a less comprehensive intervention, our implementation of the training was modified over the study period because of COVID-19. In the original design, staff from each intervention facility were trained by master stigma reduction trainers from Zambia before they delivered the training to their facility staff peers. Pre-COVID intervention facilities received a 2-day, 3-hour participatory in-person training from the master trainers, as well as several days of on-site coaching as they delivered their first round of 5-day training to their colleagues in their clinics. In the post-COVID time period, it was not possible to bring the master trainers back to South Africa, so a virtual refresher training of trainers was undertaken with the master trainers from Zambia, followed by 5-day training in the remaining S&D facilities led by the recipients of the refresher trainings and project staff. Notably, COVID modifications related to social distancing were also undertaken for the post-COVID trainings. In addition, after COVID, the staff training was not able to include all staff as intended because of other workload stresses imposed during the COVID-19 pandemic. Notably, even before COVID-19, some of the S&D facilities could not have full participation because of workload pressures. Each of these modifications (lighter training, less overall participation, and the burdens imposed by COVID-19) meant that the S&D implementation was less intensive than intended. Finally, given that the facility eligibility criteria meant that all participating facilities had to offer youth-friendly services, it is possible that the addition of the S&D training was duplicative of prior training that facility staff had already received. Further, to date the TFA has not been tested solely to focus on stigma towards AGYW seeking SRH services and thus it may require additional modifications to be appropriate for this key audience. These factors may explain why the results in the S&D reduction training arm signalled limited impact. That said, these factors do not explain why those in the S&D reduction training arm only in the longitudinal models would be less likely to use contraception than the control group; this result might reflect the emphasis of the S&D training on PrEP provision or the focus of providers on HIV prevention over other SRH needs of young women. If so, the young women in this arm may not have experienced the full effect of S&D reduction if they were coming to get contraception and still faced barriers to its adoption. Notably, in the main PrEP outcomes paper from this study,^32^ it was found that those AGYW who attended a clinic with the S&D reduction training were more likely to initiate PrEP, and the AGYW in all three intervention arms were more likely than the control group to persist on PrEP. An additional explanation for null S&D results in our study may relate to enrolment continuing during COVID in the non-S&D and control arms. The young women in these sites may have been more motivated to use a contraceptive method than those enrolled before or after COVID. This possibility is discussed in more depth below.

It is less obvious why the AGYW in the YHWC-only arm and the combined YHWC and S&D arm did not show positive effects in the longitudinal model. This may reflect the fact that this model is simply examining at each time period whether an individual is using or not. As can be seen from the data in the first outcome, there are a number of young women changing their status over the follow-up period, including about a fifth of the sample that reports being inconsistent users. Thus, when we try to examine who is using a method at each period and how this relates to programme exposure, the findings may be attenuated by the starting, stopping, and inconsistent use behaviours in the sample. Furthermore, the modelling approach used (*xtgee*) estimates population average effects, accounting for repeated measures within individuals. Thus, it is possible that the population averages shift towards null effects, diminishing the ability to detect specific relationships of the intervention conditions and contraceptive method use. By identifying the more specific contraceptive use patterns in the first analysis, we are better able to see how the intervention arms relate to being in one or another of these contraceptive use behaviour categories.

Another interesting finding relates to the positive association between enrolling in the study during COVID-19 and young women’s contraceptive adoption, continuation, and use. Those young women who enrolled during COVID might have been more motivated to avoid pregnancy and use contraception than those who enrolled before COVID. Further, those AGYW who were at home during COVID may have had increased exposure to sex by spending more time with their partners; this may have motivated them to start contraceptive methods. Our finding is consistent with studies from elsewhere in sub-Saharan Africa that have demonstrated that while there were concerns about the provision of contraception during COVID, clinics were able to ensure availability, especially once lockdowns were relaxed. For example, among adolescents in Uganda, in May 2020, as the lockdowns were relaxed, the number of contraceptive visits by adolescents ages 15–19 was higher than projected; this trend continued until the end of 2020.^38^ Furthermore, an analysis in Senegal demonstrated that while there was a drop in contraceptive adoption during the initial lockdown months, once the lockdown was relaxed, contraceptive use increased again, with a preference for longer-acting methods, including implants and intrauterine devices.^39^

This study employed a multi-component intervention that addressed individual, interpersonal, and facility-level barriers and facilitators to contraceptive use. We showed that the YWHC that addressed influences at the individual and interpersonal levels with the goal of reducing harm and increasing AGYW’s confidence and sense of power may have been an important approach, with or without the stigma and reduction training. These positive findings of the YWHC intervention on adolescent risk-taking outcomes are consistent with an earlier study where the YWHC was undertaken in Cape Town, South Africa, and the authors demonstrated reduced risk behaviours following programme exposure.^24^ Notably, as discussed earlier, the lack of effect of the S&D training may also be reflective of the selection criteria for facilities that had to offer youth-friendly services. This corresponds with a key finding from a recent scoping review of adolescent contraceptive use interventions in sub-Saharan Africa.^19^ The authors demonstrate that youth-friendly services are one of the effective intervention strategies, among others; therefore, perhaps the addition of the S&D training to facilities that already had youth-friendly services was not an important contribution to the services offered. The scoping review also demonstrated an impact of community-based programmes, mHealth, sexual and reproductive health education, counselling, and community health worker visits.^19^ The authors argue for multi-component programmes, like the one we implemented here. Notably, the national PrEP guidelines in South Africa promote the need for a minimum package of services to be provided alongside PrEP.^4^ These include pregnancy screening and contraception, along with other HIV and STI prevention services; this indicates the importance at the national level of multi-component sexual and reproductive health services. Future studies should examine how a brief empowerment intervention like the YWHC employed here can be implemented in unison with other successful SRH intervention models such as mHealth and community health worker engagement to support AGYW to meet all their sexual and reproductive health needs. As noted earlier, the lack of S&D effect may also be due in part to the pared-back implementation of the adapted Total Facility Approach, which only includes the training aspect for a limited number of staff. The TFA, originally designed to address HIV stigma, is a whole-facility approach that focuses on capacity strengthening within a facility for stigma reduction and the integration of S&D reduction into facility structures and processes.^25^ It was developed and preliminarily tested in Ghana and Tanzania and is a key component of Thailand’s integration of S&D into their National HIV response.^40^ The TFA has not been adapted and tested with a focus solely on addressing health facility stigma towards AGYW seeking SRH services. In Tanzania, the development process of the HP + HIV stigma-focused TFA added a focus on stigma towards sexually active adolescents and unmarried pregnant adolescents, demonstrating a significant decline in negative attitudes among health facility staff towards these groups.^41^ These adaptations addressing stigma towards adolescents were incorporated; however, given the lighter touch of the S&D design, this may not have led to the expected effects.

This study demonstrated the impact of the YWHC on its own and in combination with S&D reduction training; however, the study is not without limitations. The first important limitation has to do with the timing of the study, which was significantly affected by the COVID-19 pandemic. We began enrolment nearly a year before COVID; once COVID emerged and South Africa took public health measures to reduce transmission, we paused enrolment and had to adopt changes in enrolment as well as in the approach to S&D reduction training. This change meant that the S&D training in three of the six S&D training facilities was not as rigorous as it had been in the initial three facilities. Second, while the S&D training was meant to cover all staff in the S&D arm facilities, we recognise that due to strains on clinic personnel, not all staff were able to participate. This may have led to the S&D arm showing a lesser effect on contraception adoption and continuation. Third, this analysis does not include AGYW’s prior experience with stigma from clinic providers, which might be key determinants of their subsequent adoption of a method. Future studies of S&D interventions can measure reported stigma and examine whether AGYW’s differential clinic experiences over time are related to a targeted S&D training to make facilities more accessible and acceptable to them.

Additional limitations relate to availability of contraceptives and how contraceptive use is measured in this study. In particular, during the study period, there were significant stock-outs of contraceptives in South Africa,^42^ which may have affected young people’s ability to adopt and continue a method of their choice. This situation would not affect the participants in one study arm over another, but those who were already using a long-acting method (implant or IUD) might have been at an advantage and less affected by stock-outs, assuming that was their method of choice. Thus, differences by clinic arm in the methods available when a young person adopted her method (either before or during the study) would bias the results of this analysis, which focused specifically on contraception adoption, continuation, and use. In addition, contraceptive use is based on self-reporting, which may be affected by courtesy bias if young women think that they are supposed to be using a method. Finally, contraceptive adoption and use were the measure of “success” used in this study that focused on AGYW who reported that they did not want to become pregnant in the next year. This approach assumes that the young women want to be using a method and ignores the potential that they prefer to not use anything.^43^ Currently there are efforts underway to promote the use of person-centred outcomes^44^ that better capture a person’s self-defined contraceptive needs including a novel measure called preference-aligned fertility management.^45^ Application of these novel, person-centred measures to AGYW samples and to evaluations of AGWY programmes will lead to improved programmes that are truly meeting the sexual and reproductive health needs of this key population.

This study also has several strengths. First, by using longitudinal data, we could follow young women’s contraceptive practices over a 9-month period. Changes in practices were linked to the study arm that the AGYW were recruited into. Second, by working with our Community Advisory Board as well as with our Youth Advisory Board, we tailored the YWHC and S&D training to the specific needs of young women in the study communities. Third, by including biomarkers at each study visit, we were able to control for pregnancy status, which, as shown, was a key factor related to contraceptive non-use in the longitudinal models. Finally, this study had a high follow-up rate, which means that we were able to examine outcomes over the 9-month period for most of the sample.

## Conclusions

This study demonstrates that the YWHC, a brief young woman-focused, group-based empowerment intervention that reaches AGYW, can – either on its own or in combination with health facility staff S&D reduction training – lead to increases in contraceptive use among young women at risk of HIV and unintended pregnancies. Supporting young women with tailored messaging in a safe environment can increase their agency to make and act on sexual and reproductive health decisions to use (or not use) contraception and condoms; this will support the goals of SRH and HIV prevention programmes. The National Department of Health in South Africa can learn from this study and use it to expand the brief intervention for broader scale-up throughout Tshwane and Gauteng, as well as in other provinces of South Africa. Before scaling up, it will be essential to examine in more depth whether and how to improve on the YWHC and the S&D training and consider how it is incorporated into youth-friendly clinics leading to improved availability, accessibility, acceptability, and quality of sexual and reproductive health services for all adolescents. Further, it is imperative to identify which components of the YWHC and S&D training are essential for replication and which ones are flexible and adaptable for new sites. This type of implementation research will lead to a multilevel intervention that is sustainable and scalable for helping young women have the agency and autonomy to meet their health and well-being goals and avoid the risk of HIV and unintended pregnancies.

## Supplementary Material

Supplemental material. CONSORT Diagram

## Footnotes

1
https://www.gov.za/about-sa/south-africas-provinces

2
https://knowledgehub.health.gov.za/system/files/elibdownloads/2023-04/National%2520Integrated%2520SRHR%2520Policy_Final_2021.pdf

3 Most use in the sample is of modern methods that included female and male condoms, oral contraceptive pill, injectable, implant, and IUD.

4
https://www.prepwatch.org/wp-content/uploads/2020/07/South-Africa-PrEP-Guidelines_Jan2020.pdf
