# Supplementary material for: Evaluating the effects of a multi-level intervention on young women’s contraceptive use: sexual and reproductive health results of a cluster-randomised factorial-design study in Tshwane, South Africa
Source: Sex Reprod Health Matters. 2026 Jul 1;33(1):2696689. doi: 10.1080/26410397.2026.2696689 (PMC13431016; doi:10.1080/26410397.2026.2696689)
Supplement: Supplementary file 1 [file ZRHM_A_2696689_SM6537.doc]

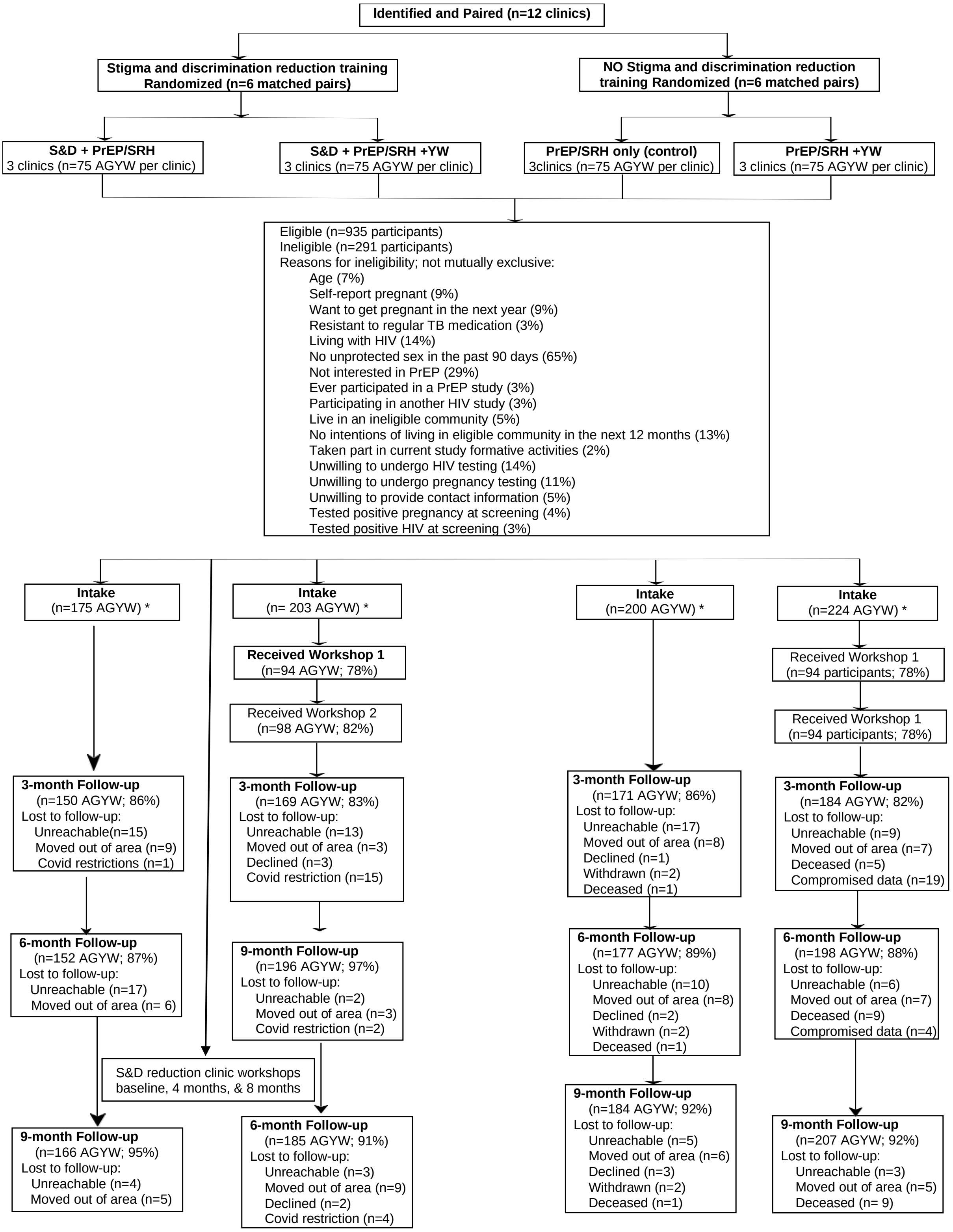


*Sample size was reduced to 50 per clinic for one clinic in each arm due to the COVID lockdown.
